# Supplementary material for: BGN/TLR4/NF-κB Mediates Epigenetic Silencing of Immunosuppressive Siglec Ligands in Colon Cancer Cells
Source: Cells. 2020 Feb 9;9(2):397. doi: 10.3390/cells9020397 (PMC7072454; doi:10.3390/cells9020397)
Supplement: Supplementary file 1 [file cells-09-00397-s001.pdf]

Supplementary Data

Table S1 Primers used in this study		
Primer		
ChIP assay		
DTDST	GCT GGG ATT ACA AGC GTG AG	TTC CCC ATC AAA GAT TCC AG
ST6GalNAc6	CCA CGA AAA ATG CTG GCT CC	CTG CTG TAG TGG TCT TGG CA
BGN exon 1 binding site	CAA ACT GCC CAG GAG TGA GT	AGG CGC AGG CTG ACT AGT TG
BGN p65 binding site	GGC CCT CTT TTT AAG GGA AG	GGT GGT TTC TCC AGG ATC AG
EZH2 p65 binding site	AGC ATA AAG CCA GAC CAG GC	TAG CTT CGC CTC TGA CGT TT
SUZ12 p65 binding site	GGT CCT TCT CTC CCC ACA AT	AGG GAA GGA GGG AGG AAA A
EED p65 binding site	AGG GGG AAG CAG GTT ACT GT	GGA CAG CGC TTC CTG ACT AT
Gene expression		
EZH2	TCA TGC AAC ACC CAA CAC TT	TTG GTG GGG TCT TTA TCC GC
SUZ12	GCA AGA ATC TCA TAG CTT GTC AGC	GCA GGA CTT CCA GGG TAA CA
EED	AGC CAA ATG TAC AAC ACT GAC T	CAG CGC CAA ATA CTG GCA TC
ST6GalNAc6	GAC GCC GGA GAG AAA TGA GT	GGG AGC CGT AAT GGA AGA CC
DTDST	CAG ATG CCT TGC TGA GTG GA	CCC ACA CCA TTA GTC CGA GG
BGN	AAC TAG TCA GCC TGC GCC T	CTC CCA GAA GCC TCT CTG CT
TLR2	TGC TGC CAT TCT CAT TCT TCT G	AGG TCT TGG TGT TCA TTA TCT TCC
TLR4	CAG AGT TGC TTT CAA TGG CAT C	AGA CTG TAA TCA AGA ACC TGG AGG

Table S1. Primers used in this study.

log2FC

-3 3

TLR4 ligands relative genes

Tissues	*ST6GalNAc6*	*SLC26A2*	*BGN*	*HSPD1*	*HMGB1*	*S100A8*	*S100A9*	*TNC*	*HYAL1*	*HYAL2*	*SDC2*	*HSPG2*	*HAS1*	*HAS2*	*HAS3*	*SFTPA1*
All	-0.768	-0.197	0.41	0.403	0.275	0.758	1.213	0.537	-1.398	ns	-0.453	0.316	-0.309	0.126	-0.351	-0.294
Adipose	ns	ns	ns	0.364	ns	ns	ns	ns	ns	ns	ns	ns	ns	ns	ns	ns
Adrenal_Gland	-0.722	ns	ns	ns	ns	ns	-1.911	ns	-0.841	-0.772	ns	ns	ns	ns	0.998	ns
Bladder	ns	ns	1.01	0.809	0.659	ns	1.886	ns	ns	0.714	ns	1.039	ns	ns	0.983	ns
Blood	ns	-0.266	0.715	0.444	0.383	0.425	0.515	0.864	ns	0.26	0.971	1.471	0.29	0.552	-0.794	0.293
Bone	ns	ns	ns	ns	ns	ns	ns	ns	ns	ns	ns	ns	ns	ns	ns	ns
Brain	-1.15	1.257	0.831	0.51	0.167	0.541	1.07	2.609	-0.764	0.813	ns	1.984	-2.01	**1.648**	0.182	-0.513
Breast	-0.648	-0.146	1.528	0.284	-0.061	1.048	1.049	ns	-1.616	-0.256	-0.539	ns	ns	-0.331	-1.063	ns
Cervix	ns	ns	ns	0.715	ns	ns	ns	ns	ns	ns	ns	ns	ns	ns	ns	ns
Colon	**-2.703**	**-3.317**	**2.274**	0.703	0.416	1.654	2.314	1.195	-0.481	0.789	0.469	0.734	0.385	0.147	ns	ns
Endometrium	-1.621	ns	0.723	0.434	ns	**1.764**	2.175	ns	ns	-0.364	-0.829	ns	ns	0.803	ns	-0.879
Esophagus	-1.323	ns	ns	ns	ns	ns	ns	ns	ns	-0.398	-1.435	-1.82	ns	ns	ns	ns
Eye	ns	ns	ns	ns	ns	ns	ns	ns	ns	ns	ns	ns	ns	ns	ns	ns
Gallbladder	ns	ns	ns	ns	ns	ns	ns	ns	ns	ns	ns	ns	ns	ns	ns	ns
Head and Neck	ns	-1.649	1.001	ns	ns	ns	ns	ns	ns	ns	1.22	1.139	ns	1.16	1.673	ns
Kidney	-0.435	0.196	0.347	-0.364	0.117	0.67	0.868	1.728	-2.024	ns	0.152	1.136	0.39	0.349	ns	**0.537**
Liver	-0.366	1.101	-1.155	ns	0.142	-0.818	ns	0.312	-1.362	0.302	ns	ns	ns	ns	ns	ns
Lung	-0.691	-0.553	0.631	0.421	-0.181	-0.821	ns	0.554	-1.385	-0.726	-0.277	-0.508	-0.421	ns	0.707	-1.375
Lymph Node	ns	ns	ns	ns	ns	ns	ns	ns	ns	ns	ns	ns	ns	ns	ns	ns
Muscle	ns	ns	ns	ns	ns	ns	ns	ns	ns	ns	ns	ns	ns	ns	ns	ns
Oral	-1.263	ns	0.969	0.711	ns	ns	ns	ns	ns	ns	ns	ns	ns	ns	1.963	ns
Ovary	ns	0.605	1.489	ns	0.222	1.032	**3.903**	1.277	-1.026	**1.282**	-0.477	**2.231**	-1.441	0.745	-0.526	ns
Pancrease	ns	-0.34	0.901	ns	0.229	-1.203	ns	ns	ns	ns	-0.751	0.879	ns	ns	ns	ns
Pharynx	ns	ns	ns	ns	0.732	ns	ns	ns	ns	ns	ns	ns	ns	ns	ns	ns
Placenta	ns	ns	ns	ns	ns	ns	ns	ns	ns	ns	ns	ns	ns	ns	ns	ns
Prostate	ns	ns	ns	ns	ns	ns	-2.556	ns	ns	-0.456	ns	ns	ns	ns	1.414	ns
Skin	-0.638	ns	0.4	0.635	ns	ns	ns	0.994	-1.177	ns	ns	ns	-0.36	ns	0.441	0.366
Small_Intestine	ns	ns	ns	ns	ns	ns	ns	ns	ns	ns	ns	ns	ns	ns	ns	ns
Spleen	ns	ns	ns	ns	ns	ns	ns	ns	ns	ns	ns	ns	ns	ns	ns	ns
Stomach	ns	ns	1.07	0.74	**0.864**	ns	ns	0.815	ns	0.697	0.62	0.816	ns	0.478	ns	-1.099
Teeth	ns	ns	ns	ns	ns	ns	ns	ns	ns	ns	**2.946**	ns	ns	ns	ns	ns
Testis	ns	ns	ns	**1.733**	ns	ns	ns	ns	ns	ns	ns	ns	ns	ns	ns	ns
Thyroid	0.341	0.374	0.838	ns	ns	0.687	1.067	2.26	-0.713	0.424	-1.126	0.295	ns	1.341	-0.514	1.31
Tongue	ns	ns	ns	0.832	ns	ns	ns	**2.791**	ns	-0.718	-1.365	-1.089	ns	ns	**2.402**	-3.068
Uterus	ns	ns	ns	ns	ns	ns	ns	ns	ns	0.732	ns	ns	**1.171**	1.322	-1.373	ns
Vagina	ns	ns	ns	ns	ns	ns	ns	ns	ns	ns	ns	ns	ns	ns	ns	ns
Vulva	ns	ns	ns	ns	ns	ns	ns	1.19	ns	ns	ns	ns	ns	ns	ns	ns

Table S2. TLR4 ligand expression level in cancers. The data were retrieved from the GENT2 database (GPL570 platform), and are only shown according to significant test results by *t*-test ($p < 0.001$). ns, not significant.

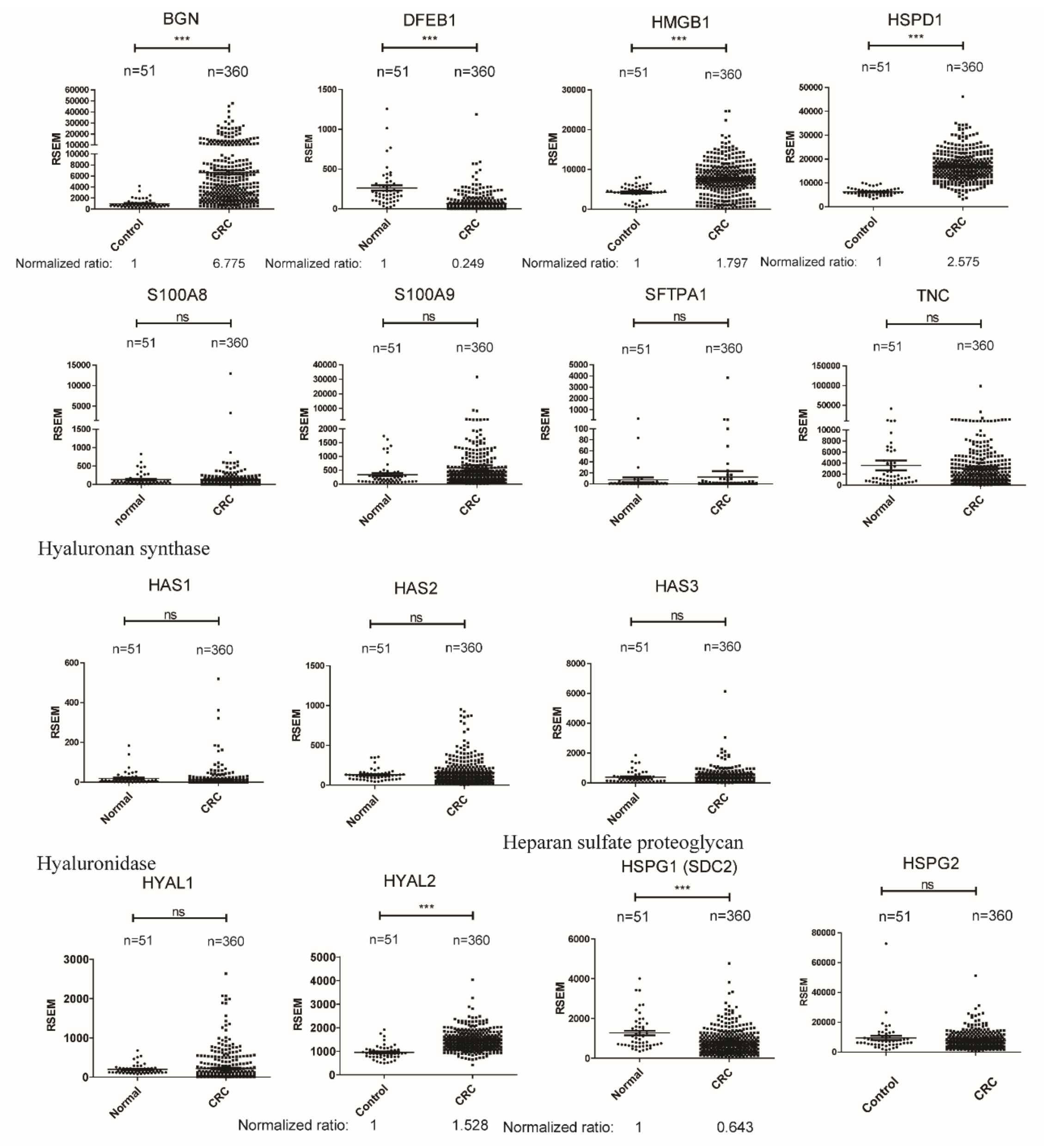

Figure S1. Large-scale RNA-Seq transcriptome analysis of TLR4 potential ligands—BGN, DEFB1, HMGB1, HSPD1, S100A8, S100A9, SFTPA1, TNC, hyaluronan synthase (HAS1, HAS2, and HAS3), and heparan sulfate proteoglycan responsive enzymes (SDC2 also called HSPG1, and HSPG2) in normal colorectal tissues and tissues from colorectal cancer from the TCGA database. n, number of patients; ***, $p < 0.001$.

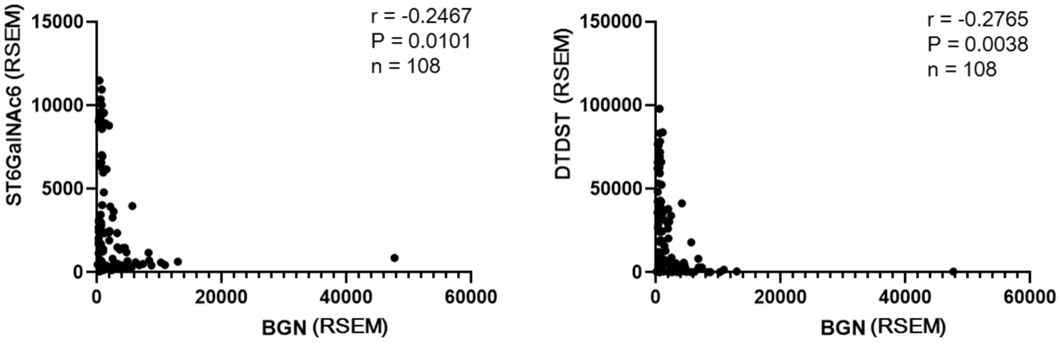

Figure S2. ST6GalNAc6 and DTDST mRNA levels were inversely correlated with BGN. Large-scale RNA-Seq transcriptome analysis of the relationship between ST6GalNAc6 and DTDST and BGN in healthy control colonic and rectal tissues and in colorectal cancer samples (stage 1) from the TCGA database. ST6GalNAc6 and DTDST each showed a significant inverse correlation with BGN mRNAs.

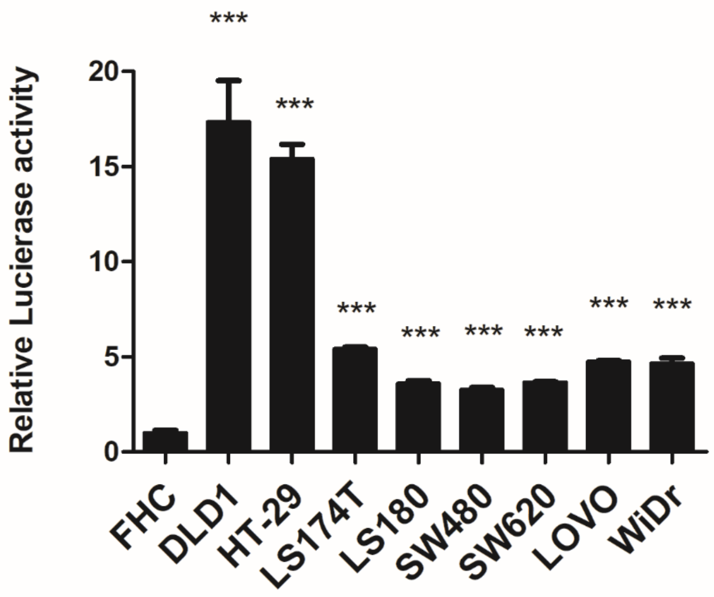

Figure S3. Relative NF-κB activity in normal intestinal cells and some colon cancer cells. Promoter analysis of NF-κB activity in normal intestinal cells—FHC—and colon cancer cells—DLD1, HT-29, LS174T, LS180, SW480, SW620, LOVO, and WiDr cells. Cells were transfected with 0.2 µg/well of the pGL4.32 (luc2P/NF-κB-RE/Hygro) vector (Promega Corporation, Madison, WI, USA), and incubated for 24 h for the subsequent dual luciferase reporter assay. Firefly luciferase activity was normalized to Renilla luciferase activity. Data are presented as the relative fold increase compared with FHC. ***, $p < 0.001$; (n = 3 with mean ± SD shown).

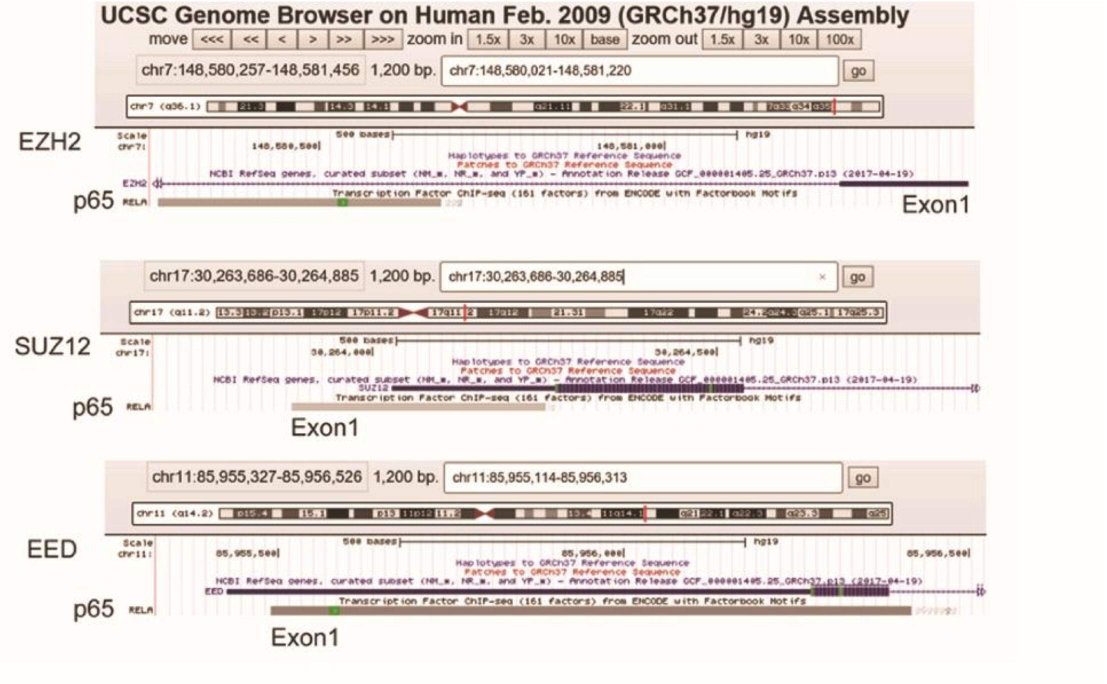

Figure S4. Identification of p65 binding regions in EZH2, SUZ12, and EED through multi-omics data analysis. Locations of p65 show gray blocker in EZH2, SUZ12, and EED. The genomic positions in this figure are based on the UCSC Genome Browser, Assembly GRCh37/hg19.

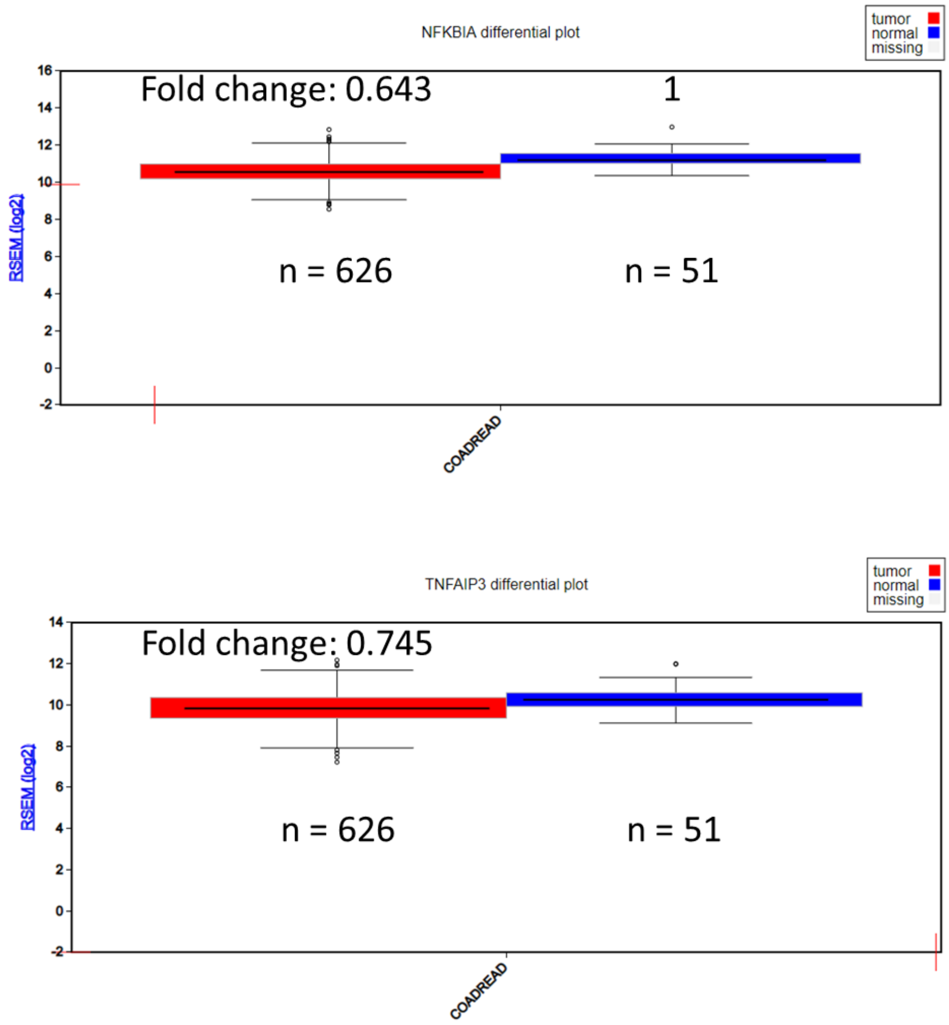

Figure S5. NFKBIA and TNFAIP3 differential plots for colorectal cancer in the TCGA database.
